# Recognition of Interaction Interface Residues in Low-Resolution Structures of Protein Assemblies Solely from the Positions of Cα Atoms

**DOI:** 10.1371/journal.pone.0004476

**Published:** 2009-02-13

**Authors:** Rupali A. Gadkari, Deepthi Varughese, N. Srinivasan

**Affiliations:** Molecular Biophysics Unit, Indian Institute of Science, Bangalore, India; Institute of Molecular and Cell Biology, Singapore

## Abstract

**Background:**

The number of available structures of large multi-protein assemblies is quite small. Such structures provide phenomenal insights on the organization, mechanism of formation and functional properties of the assembly. Hence detailed analysis of such structures is highly rewarding. However, the common problem in such analyses is the low resolution of these structures. In the recent times a number of attempts that combine low resolution cryo-EM data with higher resolution structures determined using X-ray analysis or NMR or generated using comparative modeling have been reported. Even in such attempts the best result one arrives at is the very course idea about the assembly structure in terms of trace of the Cα atoms which are modeled with modest accuracy.

**Methodology/Principal Findings:**

In this paper first we present an objective approach to identify potentially solvent exposed and buried residues solely from the position of Cα atoms and amino acid sequence using residue type-dependent thresholds for accessible surface areas of Cα. We extend the method further to recognize potential protein-protein interface residues.

**Conclusion/ Significance:**

Our approach to identify buried and exposed residues solely from the positions of Cα atoms resulted in an accuracy of 84%, sensitivity of 83–89% and specificity of 67–94% while recognition of interfacial residues corresponded to an accuracy of 94%, sensitivity of 70–96% and specificity of 58–94%. Interestingly, detailed analysis of cases of mismatch between recognition of interface residues from Cα positions and all-atom models suggested that, recognition of interfacial residues using Cα atoms only correspond better with intuitive notion of what is an interfacial residue. Our method should be useful in the objective analysis of structures of protein assemblies when positions of only Cα positions are available as, for example, in the cases of integration of cryo-EM data and high resolution structures of the components of the assembly.

## Introduction

Chemical nature and structural context of residues in a protein generate diversity in the contribution of residues towards stability and function of the protein [1]. Classifying residues as surface exposed and buried, based on their solvent accessibility, is a simple but important step towards understanding the contributions of the residues to the structural integrity [2], [3]. Surface exposed residues are often crucial for interactions with other proteins and play functional roles while the buried residues contribute more towards stability of the tertiary structure [1]. In the cellular context proteins rarely work in isolation and are often associated with other proteins to form functional assemblies. Hence, it is biologically relevant to recognize the solvent exposed regions of the assemblies and protein-protein interfaces, knowledge of which can further be made use of in the practical applications [4].

Owing to the advent of high throughput proteomic studies in combination with the computational methods, a vast amount of information is becoming available on the protein assemblies and protein-protein interaction networks [5]. However, the structural information on these huge assemblies, which indeed are the functional entities, is very sparse. The use of X-ray crystallography is often rendered limited to those assemblies that can be either purified in large enough quantities and can be reconstituted *in vitro* from the purified components. In the recent times cryo-electron microscopy has emerged as a very important technique to obtain structural information about these assemblies [6], [7]. Taking the cues from the high resolution structural studies of the individual components several successful attempts have been made to come up with the atomic level structural models of these low resolution structures, which give rough information about the protein backbone [8], [9]. However, a structure in which the atomic level models are embedded in the low resolution maps obtained from cryoelectron microscopy studies is reliable typically only upto the level of Cα trace. Uncertainty in the positions of main chain atoms and the sidechains is high. Indeed often in such modeling studies, the structures of proteins are made available only up to the Cα level. Traditionally, attempts have been made to identify the secondary structures solely from positions of Cα atoms [10], [11]. However, classification of residues as surface exposed and buried using solely the positions of Cα atoms is highly obscure as the solvent accessibility-based [12] recognition of exposed and buried residues in proteins [2], [13], [14], [15], [16], [17], [18], [19], [20] rely on the availability of side chain positions. Further, objective recognition of residues potentially in the interaction interfaces of protein-protein assemblies based only on Cα positions is also not straightforward. Such analyses are often left to visual inspection, which is highly subjective.

In the present study we first present an objective method to recognize the buried and exposed residues in the structures of proteins with positions of Cα atoms alone available. Given the reasonable success of this approach and given the importance of interactions between proteins in an assembly [21], we extended the method to recognize protein-protein interface residues solely using Cα positions. As these two proposed approaches for recognition of exposed and buried residues and interaction interface residues operate only on Cα positions this development is particularly relevant to low resolution structures of protein assemblies with atomic level structures modeled.

Interestingly in-depth assessment of our approach to identification of interaction interface residues solely from Cα positions points to structural contexts where the proposed approach identifies interface residues more effectively than the traditional approaches which use positions of other atoms such as those in the sidechains.

## Results

### Protocol

The general approach to recognize protein-protein interaction interfacial residues solely from the positions of Cα atoms mimics the popular approach used for protein-protein complex structures with all the atomic positions available and using the solvent accessibility calculations. Though there are a few criteria for identifying interfacial residues in complex structures with all the atomic positions available, in our approach based solely on Cα positions we mimic the following criterion which has been used commonly in the literature [22].

For a residue to be considered in a protein-protein interface solvent accessibility of the residue in the complex should be ≤7% and in the absence of interacting subunit the accessibility should be ≥10%.

The primary challenge in using an alteration of this criterion for complex structures with positions of only the Cα atoms available is to identify the equivalence of 7% and 10% sidechain accessibility for accessible surface area of Cα atoms as a function of the residue type.

#### Choice for the radius of the probe in the accessibility calculations on structures with Cα positions alone available

Sidechain orientation is a key factor that determines extent of solvent accessibility. Absence of sidechain positions in low resolution structures with only Cα positions available makes recognition of solvent exposed and buried residues non-trivial. However relative orientation of virtual bonds connecting contiguous Cα atoms gives a rough indication of sidechain orientation.

Our approach to recognize solvent exposed and buried residues based solely on Cα positions involves calculation of accessible surface area values of Cα using a probe sphere of appropriate radius. In this analysis we have used 1464 high resolution (≤2Å) crystal structures of proteins which are largely non-homologous with positions of all the non-hydrogen atoms available. Solvent accessibilities of all the residues in these proteins employing the standard probe radius of 1.4Å, which is commonly used for all-atom models, have been calculated. We have generated a separate coordinate dataset of only Cα atoms in these protein structures consciously deleting the coordinate data for all non-Cα atom types. We refer this dataset as “Cα-only structures”. This dataset is not entirely equivalent to a dataset of low resolution structures with only Cα positions available as the accuracy associated with Cα positions in the dataset of Cα-only structures is expected to be higher (owing to the higher resolution) than that of true low-resolution structures. However, as shown earlier [23], [24], reasonable random perturbations of Cα positions and analysis of such modified structures did not result in radically altered assignments of secondary structures.

In order to recognize the radius of the probe sphere that is appropriate for the structures with only Cα positions available we have calculated accessible surface area values of Cα atoms for the entries in the dataset of Cα-only structures using a series of probe of radii namely (in Å), 2.1, 2.5, 3.0, 3.2, 3.4, 3.5, 3.6, 3.8, 4.0. Accessible surface area (expressed in square Angstroms) of a Cα atom corresponding to a specific residue, calculated using a specific probe radius in a given protein structure, is compared to accessibility value (expressed as %) of the same residue calculated using all the available atomic positions and using a probe radius of 1.4Å. Two measures have been employed to assess the correspondence between the accessibility values and accessible surface area values.

A simple correlation coefficient has been calculated corresponding to a specific probe radius for every protein structure in the dataset of Cα-only structures. Distribution of correlation coefficients has been studied for the range of probe radii for every structure in the data set. We seek to choose the probe radius that generally provides highest correlation coefficient for most of the structures in the data set.

Rank order of the buried residue positions corresponding to the increasing order of accessible surface area of the Cα atoms for a specific probe radius is compared to the rank order of the buried residues in the same protein using all-atom model and the probe radius of 1.4Å. The parameter *per* defines the deviation in the rank correlation between the two distributions for a given probe radii:
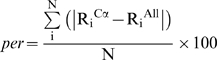



Here R_i_^All^ and R_i_^Cα^ correspond to accessibility rank of a buried residue (characterized by ≤7% solvent accessibility) from full-atom structures and ASA rank of the same residue in the Cα-only structure calculated for a specific probe radius. N corresponds to the number of buried residues.

#### Recognition of exposed and buried residues solely using Cα positions

No standard cut-off values in terms of ASA values are available to determine the buried residues solely from the positions of Cα atoms. Hence, we identified residue type dependent cut-off for accessible surface area values of Cα atoms corresponding to 7% and 10% solvent accessibility. Towards this, correlation between surface area values of Cα atoms from Cα-only records, obtained for each one of 20 residue types and the accessibility values for the same residue as obtained using the whole atom record and 1.4Å probe radius. The value of Cα accessible surface area corresponding to the 7% and 10% accessibility was then calculated from the regression lines. The ASA values obtained in such a way were then used as cut-offs to identify the residues with ≤7% accessibility and ≥10% accessibility from the Cα-only structures.

#### Recognition of interfacial residues solely from the position of Cα atoms

Having identified residue type-dependent equivalence of 7% and 10% solvent accessibility for Cα only coordinate sets it is a straightforward exercise to use the criteria of ≤7% and ≥10% to recognize interfacial residues in the protein-protein complex structures with only Cα positions available.

### Results

#### Identification of the probe radius suitable for analyzing structures at the level of Cα

For a dataset of 1464 high resolution, largely non-homologous protein structures we had calculated the percentage solvent accessibilities of residues using all atom model and the classical probe radius of 1.4Å. A dataset of Cα-only structures has been formed by deleting the positions of all the non-Cα atoms from the dataset of 1464 proteins and this dataset is referred to as “Cα-only structures”. As mentioned in the *Protocol* section various radii for the probe sphere have been used to calculate accessible surface areas of Cα atoms. Correlation coefficient has been calculated between accessibility values from full-atom models and ASA of Cα atoms in Cα-only structures for various probe radii. Table 1 lists correlation coefficients for three of the sample entries. In general, for most of the 1464 structures, the highest correlation coefficient corresponds to the probe radius of 3.5Å employed on the Cα-only structures.

**Table 1:** Correlation between the ASA values obtained for three representative Cα-only structures for various probe radii and accessibility values obtained for full-atom models and a probe radius of 1.4Å.

Probe radius	Correlation coefficient (1ah7)	Correlation coefficient (1bu8)	Correlation coefficient (1d5t)
2.1	0.550	0.513	0.46
2.5	0.630	0.584	0.57
3.0	0.690	0.657	0.65
3.2	0.718	0.662	0.674
3.4	0.720	0.663	0.685
**3.5**	**0.7212**	**0.664**	**0.687**
3.6	0.720	0.661	0.689
3.8	0.7199	0.657	0.687
4.0	0.7191	0.650	0.683

**Figure 1:**
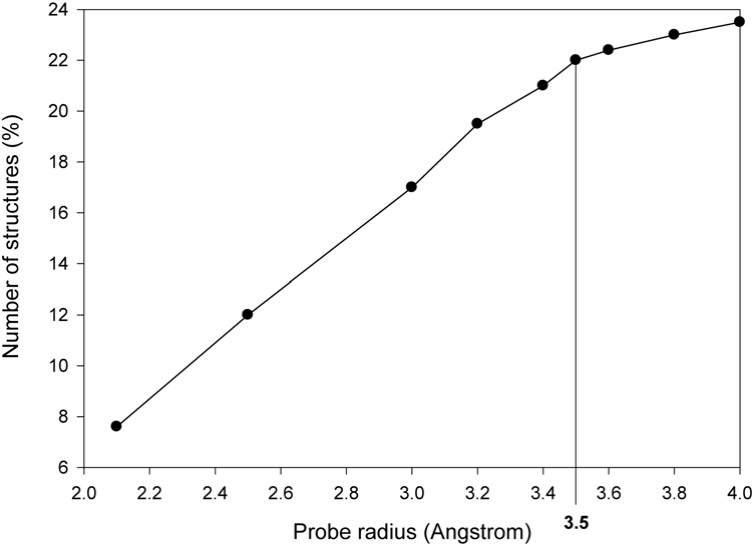
Selecting large enough probe radius for Cα-only structures. Plotted on Y-axis is the number of structures (Cα-only) showing the value of quantity “*per*” (as defined in the text) less than or equal to 20% at different probe radii values plotted on X-axis.

We have also used rank correlation of buried residues in identifying, independently, the most suitable probe radius for use with Cα-only structures. As mentioned in the *Protocol* section the parameter *per* defines the correlation between the ranks of buried residues arranged in the increasing order of percent solvent accessibilities and ranks of same residues arranged according to the ASA of Cα atoms, calculated using various probe radii, from the dataset of CA structures. Figure 1 shows the percentage number of structures that correspond to good *per* values of under 20% as a function of probe radii. It can be seen that at about 3.5Å of probe radius the number of protein structures having a good *per* value of under 20% reaches almost the maximum. Thus, from two independent analyses we identified 3.5Å as the appropriate probe radius for accessibility calculations of Cα-only structures.

#### Identification of threshold values for ASA of Cα atoms from Cα-only structures for identifying exposed and buried residues

**Figure 2:**
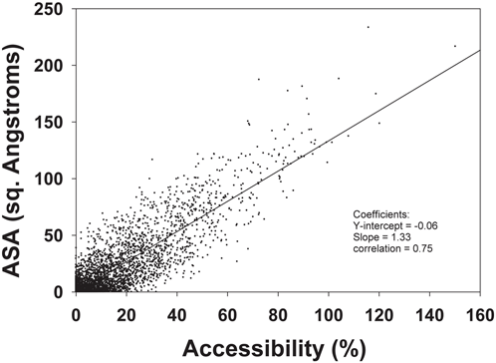
Correlation between ASA and accessibility values for Cysteine. Accessible surface area values were calculated for full-atom structures using 1.4Å probe radius and for Cα-only structures at 3.5Å probe radius. The average ASA values (obtained in case of Cα-only structures) for every residue type in each structure was then plotted against accessibility values for the same residues in each structure (as obtained using full-atom structures). Shown here are the values obtained for cysteines. The ASA values corresponding to 7% and 10% accessibility values were then computed from the regression line.

As mentioned in the section on *Protocol* for each of the 20 residue types we have analyzed the relationship between percentage solvent accessibility calculated from full-atom models using a probe radius of 1.4Å and ASA of Cα atom from Cα-only structures for a probe radius of 3.5Å. Figure 2 shows the plot for cysteine (plots for other residue types are presented in supplementary data, Figures S1, S2, S3, S4, S5). Interestingly the characteristics of regression fit varied markedly depending upon the residue type. For each of the 20 residue types we identified the ASA value at Cα corresponding to the solvent accessibility of 7% and 10%. Table 2 lists the Cα ASA values of 20 residue types corresponding to 7% and 10% sidechain accessibilities. Marked variations between Cα ASA values can be noted between various residue types. These values have been used as thresholds in identifying buried and exposed residues and also in identification of interfacial residues.

**Table 2:** The ASA values of Cα atoms for each residue type as obtained from Cα-only structures, corresponding to the 7% and 10% accessibility values from full-atom structures.

Residue	Cα ASA (Å^2^)	Cα ASA (Å^2^)
	For 7% accessibility	For 10% accessibility
Alanine	10.08	14.16
Arginine	5.64	9.54
Asparagine	8.22	12.21
Aspartate	12.8	16.85
Cysteine	9.25	13.24
Glutamate	11.56	15.61
Glutamine	7.39	11.44
Glycine	9.25	13.24
Histidine	8.23	12.31
Isoleucine	11.65	15.61
Leucine	11.51	15.47
Lysine	1.23	5.37
Methionine	11.49	16.02
Phenylalanine	12.14	15.95
Proline	9.96	13.04
Serine	9.25	13.24
Threonine	9.25	13.24
Tryptophan	11.61	15.63
Tyrosine	1.47	5.16
Valine	10.77	14.82

#### Assessment of the accuracy of recognition of buried residues from Cα-only structures using ASA cutoff

Using an independent data set of 1100 high resolution protein structures, we have recognized buried and exposed residues using the positions of Cα atoms only and using the thresholds defined for each of the 20 residue types. The buried and exposed residues thus identified were assessed by calculating sensitivity and specificity values for the two classes of the residues namely buried and non-buried (exposed), and the overall accuracy as well as the correlation coefficient using the expressions given in the *Methods* section. Table 3 summarizes the average values for these parameters for the set of 1100 structures. The sensitivity of the buried residues (*Sens_bur*) can be defined as the number of buried residues identified out of the total number of actual buried residues while the specificity as the actual number of true buried residues out of the total number of the residues that have been identified as the buried residues. As indicated in the Table, for the heterogeneous dataset that has been used here, the method recognized the buried residues with significantly high accuracy of about 85%. It has covered about 90% of the buried residues out of total number of buried residues. For any method while it is very important to correctly recognize the positives, it is equally important (sometimes even more important) to recognize the negatives correctly. Hence, we defined the sensitivity and specificity values in terms of non-buried (exposed) residues as well. The sensitivity of the exposed residues then can be defined as the number of residues identified as exposed residues from the total number of actual exposed residues. The specificity is defined as the actual number of exposed residues out of the total number of residues identified as exposed residues. As can be seen from the correlation, sensitivity, specificity and accuracy values listed in Table 3 our approach to identify exposed and buried residues has worked remarkably well. Thus, from the Cα positions and with sidechain positions unavailable, it is possible to get a good idea about exposed and buried residues. Before extending the proposed approach, which seems to work reasonably well with identification of exposed and buried residues, to protein-protein complexes we compared the performance of proposed method with results of solvent accessibility calculation on the all-atom models generated starting from Cα positions.

**Table 3:** The average values of the validation parameters calculated for the dataset of 1100 structures.

Accuracy	Correlation coefficient	Sensitivity(buried)	Specificity(buried)	Sensitivity (exposed)	Specificity (exposed)
0.84±0.08	0.66±0.19	0.89±0.09	0.67±0.13	0.83±0.06	0.94±0.01

#### Comparison of the performance of the proposed method with the results from models of all-atoms generated staring solely from Cα positions

An alternate approach to identifying solvent exposed and buried residues starting solely from Cα positions is to generate all atom models from Cα trace and employ the traditional solvent accessible surface area calculations on the dataset of coordinates of all the atoms in the proteins. For this purpose we have employed two methods to generate positions of sidechain atoms: the sidechain modeling approach employed by Sali and Blundell in their comparative modeling software MODELER [25], [26] and the approach proposed by Dunbrack and coworkers [27] encoded in the Scwrl3 software. The consolidated results obtained for a sub-dataset involving randomly selected 20 proteins are summarized in Table 4. The sensitivity and specificity for exposed residues derived from all-atom models generated from Cα positions are better than the results for buried residues. However it is clear from the table that all the sensitivity and specificity values are better for the proposed approach which involves no modeling of sidechain positions. Performance of the newly developed method is clearly better particularly in terms of correctly identifying buried residues. The overall sensitivity and correlation-coefficient are also markedly better for the proposed approach than the ones for all-atom models generated from Cα positions. Perhaps, the error introduced in side chain predictions/modeling is carried forward in the recognition of buried residues, which is successfully avoided in the proposed approach by calculating accessible surface areas for Cα positions only.

**Table 4:** Comparison of performance of recognition of exposed and buried residues using proposed Cα-based approach, all-atom models generated starting from Cα positions in relation to all-atom crystal structures.

Method	Accuracy	Correlation coefficient	Sensitivity (buried)	Specificity (buried)	Sensitivity (exposed)	Specificity (exposed)
Cα only	0.97	0.7	0.88	0.69	0.87	0.96
MODELLER	0.84	0.27	0.38	0.46	0.87	0.82
Scwrl3	0.87	0.34	0.4	0.5	0.91	0.86

The structures with Cα atom positions only were subjected to side chain modelling using two different methods (MODELLER and Scwrl3). Solvent accessible and buried residues were subsequently identified using these all-atom models and the proposed method (Cα only) were compared with results from using the all-atom crystal structures.

Having obtained these encouraging results, the method was then further extended to recognize the residues in the interface of protein-protein complexes.

#### Recognition of interface residues

Interface residues have been recognized for a high resolution dataset of 1100 protein-protein complex structures using the accessibility criteria mentioned in an earlier section. The residues were tagged as the interface residues if the accessibility values in complex form were less than or equal to 7% and in the isolated chain the accessibility value of the same residue increases to greater than or equal to 10%. In case of the Cα-only structures of the protein-protein complexes the ASA cutoff values corresponding to the above mentioned accessibility cutoffs were calculated for each amino acid as mentioned previously (Figure 2). The interface residues were then identified using these accessible surface area cutoffs (Table 2).

As mentioned previously in case of the buried residues, to validate the results obtained in case of the Cα-only structures the sensitivity and specificity values were calculated for two classes of the residues namely interface and non-interface residues. Also, the accuracy and the correlation coefficient values were calculated using the formulas mentioned in the *Methods* section. Table 5 summarizes the average values of these parameters calculated for the dataset of 1100 structures. Although the Cα-only structures lacked side chain information, the values of the parameters mentioned above clearly indicate that the interface residues could now be identified solely from Cα positions with high accuracy. The method performs extremely well in identification of the non-interface residues. In determining the interface residues, keeping in mind the limited structural information available, method performed significantly well.

**Table 5:** The average values of the validation parameters calculated for the dataset of protein-protein complexes in the recognition of interface residues solely from Cα positions.

Accuracy	Correlation coefficient	Sensitivity (interface)	Specificity (interface)	Sensitivity (non-interface)	Specificity (non-interface)
0.94±0.04	0.58±0.19	0.7±0.23	0.58±0.23	0.96±0.03	0.94±0.04

#### Assessment of false positives

**Figure 3:**
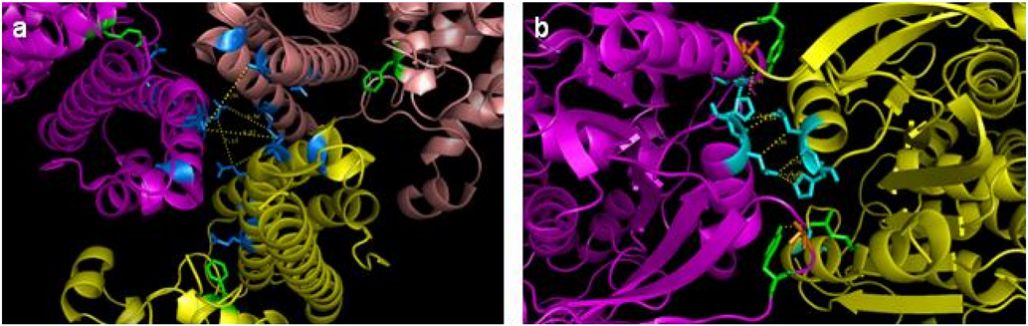
Visual validation of the interface residues determined using Cα-only records. Visual graphics tool Pymol was used to visualize the interface residues as determined in case of Default structures as well as Cα-only structures. Shown here in Figure 3a is the PDB structure of 1l7a (Cephalosporin C deacetylase) and in Figure 3b 2fef (Protein PA2201 from Pseudomonas aeruginosa), wherein the interface residues are shown in sticks while the remaining structure as cartoon. The interface residues determined using whole ATOM record are shown in green color while those determined using Cα-only records are shown in blue/cyan color. The residues in orange are the common residues between the two.

A few residues were identified as interface residues while apparently they are not interfacial residues. Hence, the apparent false positive residues were further looked at more closely. The visual inspection of these residues in Pymol [28] revealed that the residues may not be the actual false positive residues. Figure 3 illustrates a couple of such cases. From these figures it can be seen that the residues identified as interfacial only in our method using Cα positions seems truly in the interface interacting with the associated protein.

It is possible that residues in the periphery of the interface with solvent accessibility values greater than 7% even in the complexed form interact with the associated protein. These residues may not be considered as interfacial residues due accessibility values greater than 7% in the complexed form. Our method based solely on Cα positions capture these cases successfully despite the absence of sidechain positions.

Further these “false positives” were found to be fairly conserved in the course of evolution (data not shown) reinforcing the important role of these residues in the formation of protein-protein interaction interface.

#### Assessment of the method involving distance based approaches to recognize interfacial residues

Apart from accessibility based method there are several other methods [29] that are used routinely for protein-protein interface identification namely radial cutoff method [30], Voronoi polyhedra-based method [31], distance based method [32] etc. or Half sphere exposure method [33] to measure solvent exposure of residues and thus to determine interface residues. Methods such as radial cutoff and half sphere exposure require prior knowledge of Cβ positions hence in absence of it modeling becomes essential. Considering the results obtained in case of determination of buried residues subsequent to modeling side chain positions (Table 4), we have assessed our approach to identify protein-protein interfacial residues by comparing it with distance-based method applied to the original crystal structures of complexes. Towards this, 20 oligomeric structures were selected randomly from the original protein-protein complex dataset. The interface residues for the particular subunit was then determined using the following distance criterion: if the distance between the two atoms across the interacting subunits is less than or equal to the sum of their van der Waals radii plus 0.5Å [32] then the residues involving these two atoms are considered to be interacting and also considered to be in the interface. The interface residues obtained this way from the crystal structures were then compared with those recognized using the proposed approach which uses Cα positions only. In order to account for the differences in the two methods of interface determination the results of interface determination using distance based method were also compared with those determined using accessibility criterion with all-atom record from the crystal structures. Table 6 summarizes the results. The results clearly indicate that, in terms of various sensitivity, specificity, accuracy and correlation coefficient parameters, the extent of correspondence between the proposed approach (using Cα positions only) and distance-based approach based on crystal structures is highly similar to the correspondence between results of solvent accessibility of all-atom (crystal) structures and distance-based approach which is also based on crystal structures. The modest correspondence between results from solvent accessibility of all-atom structures and distance-based approach is a reflection of different ways of defining protein-protein interaction interface. Thus, seemingly less sensitivity values observed here can be attributed to the differences in two criteria of interface determination rather than they being the shortcomings of the newly developed method.

**Table 6:** Comparison of interface recognition using the proposed approach (Cα), solvent accessibility calculations on all-atom crystal structures (ASA) and inter-subunit distance calculation using crystal structures (Distance).

Method	Sensitivity interface	Specificity interface	Sensitivity non-interface	Specificity non-interface	Accuracy	Correlation coefficient
Cα *versus* Distance	0.37	0.86	0.99	0.84	0.86	0.5
ASA *versus* Distance	0.34	0.92	0.99	0.83	0.85	0.49

#### Identification of interface residues in low resolution protein complexes

A set of protein structures at low resolution was considered with only Cα positions available (Table 7) and the interface residues were recognized using our approach. Analysis of multiple sequence alignments of these protein components suggest that, in general, the residues recognized to be in the interface are conserved or conservatively substituted better than the solvent exposed residues in the complex (data not shown). Further, the manual analysis of these low-resolution structures suggests the strong possibility of the residues recognized as interfacial are actually present in the interface. The list of interfacial residues recognized in these structures are listed in supplementary Table S1. As can be observed in the table, not all the chains in the assembly contribute equally in the interface formation, although in many cases they are equivalent in their primary structure (amino acid sequence as in case of homo multimers).

**Table 7:** List of low resolution structures used with only Cα positions available.

PDB ID	Description
1ffk	Ribosomal unit
2akh	SECYEG ribosomal unit
2esg	Complex of IgA and serum albumin
2bcw	Ribosomal protein
1xi4	Clathrin coat

## Discussion

An approach has been developed to identify the buried and exposed residues in proteins solely based on the positions of Cα atoms. As shown using a large number of protein structures with complete atomic positional entries available the method works with very good accuracy, sensitivity and specificity. It is interesting to note that specificity, sensitivity, accuracy and correlation of the results of proposed method is better than that of all-atom models generated starting solely from Cα positions. Aside, the proposed method does not involve the otherwise additional step of sidechain modeling in order to identify solvent exposed and buried residues solely from Cα positions.

The approach has been extended to recognize residues in the protein-protein interfaces. Assessment of the performance reveals that the proposed method works well. In fact the structural roles of residues those are recognized as interfacial in our approach, but not in the approach using full-atom model suggest that our approach is useful even if the complex structure has positions of all the atoms available. The proposed approach seeks to mimic the solvent accessibility-based identification of protein-protein interface as applied to all-atom structures. The extent of agreement between the results of proposed approach and inter-subunit distance-based approach is a reflection of difference in perceptions and definition of protein-protein interfacial residues.

The proposed method is highly relevant in the analysis of low resolution structures with only the Cα positions available. Our work has a specific impact on the emerging low resolution pictures of fundamentally important protein assemblies obtained by embedding atomic resolution structures in cryo-EM maps. Results of our approaches employed on such structures should highlight the fundamental principles of stability and specificity of multi-protein assemblies and evolution of such complexes.

## Methods

### Datasets and the programs used

The two different datasets have been used in the present study namely a set of 1464 high resolution structures (comprising monomers) and a set of 1100 structures of protein-protein complexes. These datasets were culled using PISCES [34] for resolution ≤2Å, maximum percentage identity being 25% and maximum R-value being 0.3. The present study was initiated with the aim of determining the surface exposed residues from the Cα records in the low resolution structures. Hence, from the atomic coordinate files in the PDB format, Cα records were extracted. Thus, every PDB structure has been represented in two versions namely the one with whole ATOM record and the second one with only Cα records (will be referred to as Cα-only structures).

NACCESS [35] program has been used to calculate the accessible surface area and accessibilities.

### Performance measures

Performance of the method was measured by calculating the following parameters;

**Sensitivity (buried) or Sensitivity (interface) = TP/(TP+FN)**
**Specificity (buried) or Specificity (interface) = TP/(TP+FP)**
**Sensitivity (exposed) or Sensitivity (non-interface) = TN/(TN+FP)**
**Specificity (exposed) or Specificity (non-interface) = TN/(TN+FN)**
**Accuracy = (TP+TN)/N**
Correlation Coefficient = ((TP*TN-FP*FN)/(sqrt((TP+FN)(TP+FP)(TN+FP)(TN+FN))))

Where TP : True positives; FP : False positives; TN : True negatives and FN : False negatives.

## Supporting Information

Figure S1 Accessibility plots for Aspargine, Glutamine, Aspartate and Glutamate
(101.02 MB TIF)

Figure S2 Accessibility plots for Alanine, Valine, Leucine and Isoleucine
(101.02 MB TIF)

Figure S3 Accessibility plots for Phenylalanine, Tyrosine, Tryptophan and Methionine
(101.86 MB TIF)

Figure S4 Accessibility plots for Lysine, Arginine, Histidine and Proline
(101.02 MB TIF)

Figure S5 Accessibility plots for Serine, Threonine, Cysteine and Glycine
(101.67 MB TIF)

Table S1 supporting information table
(0.04 MB DOC)
