# Supplementary material for: Recognition of Interaction Interface Residues in Low-Resolution Structures of Protein Assemblies Solely from the Positions of Cα Atoms
Source: PLoS One. 2009 Feb 13;4(2):e4476. doi: 10.1371/journal.pone.0004476 (PMC2641018; doi:10.1371/journal.pone.0004476)
Supplement: Table S1 — supporting information table (0.04 MB DOC) [file pone.0004476.s006.doc]

**Table S1**

**Interface residues recognized for 1ffk complex:**

Residue No (as in PDB)

A chain

ILE 45

PRO 54

GLU 72

GLY 75

VAL 76

PRO 90

VAL 103

GLY 157

VAL 158

GLY 161

GLY 162

B chain

HIS 50

VAL 52

GLU 66

THR 67

E chain

THR 37

GLU 57

GLU 58

MET 61

G chain

LEU 105

H chain

VAL 88

PHE 90

PRO 112

I chain

MET 4

ILE 8

VAL 20

LEU 23

K chain

LEU 27

N chain

PRO 26

VAL 30

P chain

VAL 10

R chain

GLY 16

VAL 22

S chain

ALA 32

W chain

SER 53

ILE 54

ILE 64

ALA 65

GLY 66

GLY 67

ALA 68

THR 73

GLY 76

VAL 79

Y chain

ALA 12

**Interface residues recognized for 2akh complex:**

A chain

LEU 10

ALA 13

VAL 17

GLY 47

GLY 49

THR 53

LEU 58

LEU 62

ILE 65

ILE 66

B chain

GLN 4

ASP 8

ALA 29

ARG 34

GLY 36

ILE 77

THR 101

LEU 102

GLU 104

ILE 105

ARG 114

GLN 118

THR 159

THR 166

GLY 167

PHE 170

TRP 173

LEU 174

GLN 177

PHE 192

ALA 193

ALA 197

VAL 235

GLY 240

LEU 316

ALA 322

ILE 326

PHE 327

PHE 330

VAL 376

ILE 413

C chain

VAL 21

VAL 22

LEU 25

ALA 29

GLY 32

ALA 55

GLY 56

LEU 60

THR 63

GLY 65

PHE 71

THR 101

TRP 109

ASP 112

VAL 116

X chain

VAL 7

LEU 10

ALA 13

VAL 17

MET 21

LEU 22

GLN 24

LEU 42

ASN 50

PHE 51

THR 53

MET 55

LEU 58

LEU 62

ILE 65

ILE 66

Y chain

PRO 5

SER 11

ALA 29

LEU 30

VAL 32

ARG 34

GLY 36

ILE 77

ILE 92

THR 96

ALA 103

ILE 116

GLY 167

MET 169

PHE 170

TRP 173

LEU 174

GLN 177

ALA 197

VAL 235

ARG 372

VAL 376

GLY 377

ILE 381

VAL 410

MET 414

Z chain

VAL 79

THR 101

TRP 109

ASP 112

VAL 116

**Interface residues recognized for 2bcw complex:**

A chain

SER 65

PHE 66

THR 67

PHE 68

B chain

ARG 73

GLY 74

GLU 104

LYS 107

GLU 111

GLU 116

VAL 119

C chain

ALA 221

MET 228

LEU 232

**Interface residues recognized for 2esg complex:**

A chain

TRP 48

SER 62

TYR 96

GLU 108

TRP 112

GLY 115

LEU 133

SER 134

LEU 135

GLN 139

VAL 145

LEU 149

PHE 175

SER 189

GLN 191

CYS 245

HIS 353

LEU 355

PRO 357

GLU 361

THR 369

THR 371

LEU 373

ALA 415

THR 417

ILE 419

SER 464

VAL 470

ASP 471

THR 473

CYS 474

TYR 475

B chain

TRP 48

SER 62

TYR 96

GLU 108

TRP 112

GLY 115

LEU 133

SER 134

LEU 135

GLN 139

VAL 145

LEU 149

PHE 175

SER 189

GLN 191

CYS 245

ALA 292

GLN 294

HIS 353

LEU 355

PRO 357

GLU 361

THR 369

THR 371

LEU 373

ALA 415

THR 417

ILE 419

C chain

VAL 77

LEU 80

L chain

ALA 34

LEU 46

PHE 91

PRO 95

LEU 96

PHE 98

PHE 116

PHE 118

PRO 119

PRO 120

VAL 133

LEU 135

THR 164

SER 174

SER 176

THR 178

M chain

ALA 34

LEU 46

PHE 91

PRO 95

LEU 96

PHE 98

PHE 116

PHE 118

PRO 119

PRO 120

VAL 133

LEU 135

THR 164

SER 174

SER 176

THR 178

**Interface residues recognized for 1xi4 complex:**

A chain

LEU 845

VAL 849

ARG 852

ARG 854

TRP 861

ILE 866

HIS 867

GLU 868

GLU 896

LEU 1283

TYR 1290

MET 1302

ALA 1306

LEU 1309

ARG 1311

ALA 1355

GLU 1475

LEU 1504

GLU 1584

TRP 1587

B chain

ARG 444

ALA 1355

GLU 1475

LEU 1504

GLU 1584

TRP 1587

ASP 1614

C chain

LEU 820

GLU 826

LYS 830

ILE 833

ARG 837

ASN 1248

PHE 1258

VAL 1261

GLN 1270

LEU 1274

ILE 1276

VAL 1277

VAL 1278

HIS 1279

ALA 1355

GLU 1475

LEU 1504

GLU 1605

D chain

THR 842

LEU 845

GLU 848

VAL 849

ARG 852

ARG 854

TRP 861

GLU 868

GLU 1282

LEU 1283

LEU 1286

TYR 1290

MET 1302

ALA 1306

E chain

GLY 443

ARG 444

LEU 820

GLU 826

LYS 830

ILE 833

ARG 837

ASN 1248

VAL 1261

GLN 1270

GLY 1273

LEU 1274

ILE 1276

VAL 1277

VAL 1278

HIS 1279

ALA 1355

PHE 1414

GLU 1475

LEU 1504

GLU 1584

TRP 1587

F chain

LEU 845

GLU 848

VAL 849

ARG 852

ARG 854

TRP 861

GLU 863

GLU 868

GLU 896

VAL 1277

GLU 1282

LEU 1283

LEU 1286

TYR 1290

MET 1302

ALA 1306

PHE 1414

GLU 1475

LEU 1504

G chain

LEU 820

LYS 830

ILE 833

ARG 837

ASN 1248

PHE 1258

VAL 1261

PHE 1266

GLN 1270

GLY 1273

LEU 1274

ILE 1276

VAL 1277

VAL 1278

HIS 1279

ALA 1355

PHE 1414

GLU 1475

LEU 1504

GLU 1584

TRP 1587

ASP 1611

H chain

THR 842

LEU 845

GLU 848

VAL 849

ARG 852

ARG 854

TRP 861

GLU 868

LEU 1283

LEU 1286

TYR 1290

MET 1302

ALA 1306

ALA 1355

PHE 1414

GLU 1475

LEU 1504

MET 1596

I chain

ALA 1355

PHE 1414

GLU 1475

LEU 1504

GLU 1584

TRP 1587

ILE 1591

ASP 1611

ASP 1614

SER 1618

J-R chains

ASN 155
